# Increasing Rate of Pulmonary Embolism in Trauma Patients at a Level One Trauma Center: An Opportunity for Quality Improvement?

**DOI:** 10.7759/cureus.21793

**Published:** 2022-01-31

**Authors:** Jeremy V McDuffie, Michelle L Medintz, John T Culhane

**Affiliations:** 1 Department of Trauma Surgery, Saint Louis University School of Medicine, St. Louis, USA

**Keywords:** txa, tranexamic acid (txa), massive transfusion protocol, multidetector computed tomography (ct) pulmonary angiography (ctpa), acute care surgery and trauma, deep venous thrombosis (dvt), pulmonary embolism (pe)

## Abstract

Introduction

Pulmonary embolism (PE) is the most common cause of preventable hospital death in trauma patients, with 100,000 patients dying from PE annually. A steadily increasing PE rate was observed over seven years in the trauma population at a single level one trauma center. Our study seeks to analyze this trend by examining risk factors and searching for targets for improvement. We hypothesized that a change in one or more modifiable risk factors was associated with the increased PE rate.

Methods

This retrospective cohort study considered trauma patients admitted to our trauma center between 2012 and 2018. The change in PE rate over time and correlation with various risk factors were examined using logistic regression. The study population was divided into two cohorts: early (2012-2015), and late (2016-2018). Data were collected from a prospectively maintained trauma database. More detailed information was obtained from individual patient charts for 533 patients worked up for PE. Risk factors were evaluated using both univariate and multivariate analysis.

Results

A total of 14,986 trauma patients were included in the study, of which 132 were diagnosed with PE. The PE rate was 1.11% in the late group compared to 0.67% in the early group (p=.004). We detected no association between the PE rate and preventive measures such as screening for and treating deep venous thrombosis (DVT), placing inferior vena cava (IVC) filters, and patterns of chemical DVT prophylaxis. We did not observe a distal migration of the anatomic distribution of PEs on CT pulmonary angiogram (CTPA). There were nonsignificant trends between PE rate and changes in population demographics and injury patterns, increased frequency of major surgery, and increased tranexamic acid (TXA) use. Of known risk factors for PE, units of packed red blood cells (PRBC) (p=0.041), units of fresh frozen plasma (FFP) (p=.037), and the number of patients receiving transfusion (p=0.043) were all significantly greater in the later period.

Conclusion

Change in hemostatic resuscitation practices (use of balanced ratios of blood products) is most likely to have contributed to the increased PE rate at our institution. However, PE in trauma is multifactorial, and the increased rate cannot be attributed to any single factor. We did not observe a lapse in preventive measures commonly considered indices of quality of care. Caution is advised against overreliance on PE rate as a measure of quality.

## Introduction

Pulmonary embolism (PE) remains an important cause of mortality in hospital inpatients [1]. With an estimated 100,000 patients dying from PE in the United States annually, prevention has long been a point of focus for all providers [2]. It is especially a concern in trauma since both injuries themselves and the major surgery often required to treat injuries are considered risk factors for PE [3]. A 2017 study cites a 0.6% PE rate reported by the American College of Surgeons Trauma Quality Improvement Program (TQIP) for patients with an Abbreviated Injury Scale (AIS) of at least three in at least one body region [4]. PE is the third most common cause of death among trauma patients who survive the initial 24 hours [1]. 

PE is also considered the most common cause of preventable hospital death [5]. When examining the PE rate of a given hospital, this raises the question of how well preventive measures are implemented, and whether the PE rate should be considered a surrogate for overall quality. In October 2008, the Centers for Medicare and Medicaid Services (CMS) decided that PE could be avoided with use of evidence-based guidelines and stopped reimbursing hospitals for hospital-acquired PE in addition to certain other complications deemed preventable [6]. Hospital venous thromboembolism (VTE) rates are publicly reported, which can affect a hospital’s reputation in the community along with influencing referral patterns and patients’ choice of facility [7].

We present an analysis of a trend of increasing PEs in a level one trauma center, with examination of risk factors both modifiable and non-modifiable. Our goal is to identify changes in patient risk and practice patterns that may influence PE rate, and to search for potential targets of improvement. In doing so, we hope to identify patterns that may be generalizable to other trauma centers. Our hypothesis is that one or more modifiable risk factors has changed in our institution, increasing the risk of PE.

## Materials and methods

This is a retrospective cohort study designed to investigate an observed increase in the incidence of PE over time in a level one trauma center. The change in PE rate versus time was evaluated for significance with a logistic regression. For ease of analysis and to create two groups containing approximately equal numbers of participants, the patients were divided into two cohorts: early (2012-2015) and late (2016-2018). The incidence of risk factors was compared between the early and late periods. Demographic and injury characteristics were queried from a prospectively maintained trauma database. This institutional database contains elements submitted to TQIP in addition to other data selected for institutional use. For some of the variables, the registry was not sufficiently granular. To retrieve more detailed information, we did an in-depth chart review of the subset of 533 patients who were worked up for PE.

To better estimate risk, we calculated the Greenfield Risk Assessment Profile for each patient [8]. Due to limitations of the current data set we modified three categories. The registry does not record whether the patient had a femoral catheter, therefore this variable is omitted. For greater than four transfusions in 24 hours, we used transfusion of fresh frozen plasma (FFP) as a surrogate, because this is part of our massive transfusion protocol (MTP).

Chi square was used to test for significance for categorical variables. Mann-Whitney U test was used to test for significance for ordinal variables. T-test and Mann-Whitney U test were used to test for significance for continuous variables based on the sample size and distribution. Multivariate analysis of potential predictors of PE was performed with logistic regression. All statistical analysis was conducted with SPSS Statistics, version 26.0 (IBM Corp., Armonk, NY, USA). A p value <0.05 was considered significant.

## Results

In summary, the tables show that the PE rate increased steadily over the seven-year period from 2012 through 2018. When this time span was divided into early and late periods, the PE rate, but not the deep venous thrombosis (DVT) rate, was significantly higher in the late versus early period. The anatomic distribution of PE did not change significantly, thus no distal migration of PE in the pulmonary vasculature was observed. On univariate analysis, variables significantly associated with PE were higher injury severity score (ISS) and Greenfield Score, pelvic fractures, severe lower extremity, abdominal, and thoracic injury, transfusion of blood components, and massive transfusion. Known risk factors for PE significantly increased in the later period include age, units of packed red blood cells (PRBC) and FFP, severe abdominal injury, severe vascular injury, pelvic fracture, major surgery, any blood product transfusion, and massive transfusion. Significant predictors of PE on multivariate analysis are years after 2012, severe lower extremity and thorax injury, major surgery, and any transfusion of blood products.

**Figure 1:**
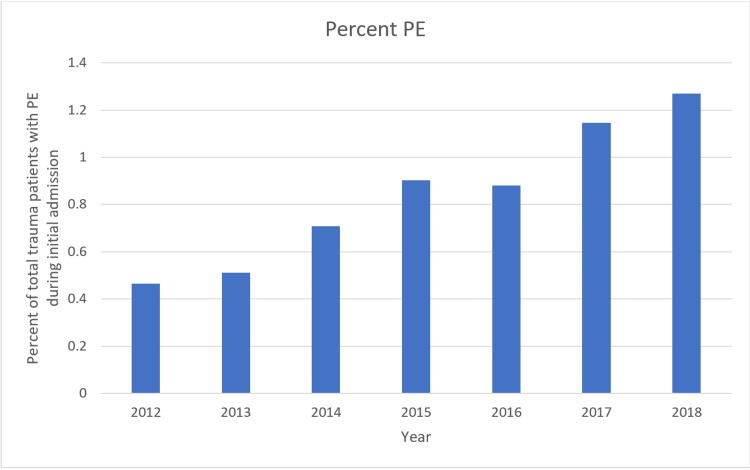
Rates of pulmonary embolism (PE) over time.

There were 14,986 trauma admissions from June 2012 to December 2018. A total of 132 patients were diagnosed with PE (Figure 1). One hundred and thirty-one were diagnosed with DVT. Twenty-one patients had DVT and PE. Five hundred and eight patients underwent CT pulmonary angiogram (CTPA). Forty-three additional patients had a PE diagnosed incidentally on CT during initial trauma workup. These were not excluded from the study. Mortality was five (3.3%) among patients who had PE, and 745 (4.3%) among patients who did not have PE (p=0.55). Twelve patients worked up for PE had bleeding complications, of whom one died.

 Table 1 depicts the rates of PE, DVT, and PE workup between patients seen at our institution during the early and late periods.

**Table 1:** Rates of PE, DVT, and PE workup in early versus late period. PE: pulmonary embolism; DVT: deep venous thrombosis; CTPA: CT pulmonary angiogram.

	Early		Late		
	n	%	n	%	p
PE	51	0.67	81	1.11	0.004
DVT	63	0.82	68	0.93	0.48
Patients Undergoing CTPA Protocol	238	3.1	270	3.69	0.048
PE Protocol Positive Rate	46	19.33	61	22.59	0.37
Incidental PE	23	0.3	19	0.26	0.64

Table 2 reveals the anatomic distribution of PEs identified with CTPA in the early versus late periods. 

**Table 2:** Anatomic distribution of pulmonary embolism (PE) in early versus late period.

	Early		Late		
	n	%	n	%	p
Proximal	13	25.5	19	23.5	0.79
Segmental	30	58.8	47	58	0.93
Subsegmental	15	29.4	20	24.7	0.55
Isolated Subsegmental	11	21.6	16	19.8	0.8
Bilateral	19	37.3	20	24.7	0.12

Table 3 and Table 4 show the analysis of differences in demographics, injury patterns, and treatment practice between patients found to have PE and those found to not have PE. 

**Table 3:** PE incidence classified by continuous risk factors for PE. Table 3 notes the mean number of units of products received by patients found to have PE versus patients who did not have PE. * Comorbidities reported in the trauma registry. ** Risk factors of PE in the subset of patients who underwent imaging for PE. PE: pulmonary embolism; BMI: body mass index; ISS: injury severity score; PRBC: packed red blood cells; FFP: fresh frozen plasma.

	Had PE		Did Not Have PE		
	n	mean	n	mean	p
Age	131	46.8	14747	46.5	0.85
BMI	128	31	13259	28.7	0.18
ISS	131	21.4	14744	11.7	< .001
Number of Comorbidities*	132	0.7	14854	0.79	0.3
Greenfield Score	132	11.5	14854	5.6	< .001
PRBC**	132	3.33	401	2.31	0.14
FFP**	132	1.44	401	1.09	0.48
Platelets**	132	0.66	401	0.65	0.98
Days Without Chemical Prophylaxis**	132	3.48	398	3	0.06

**Table 4:** Pulmonary embolism (PE) incidence classified by categorical risk factors for PE. * Risk factors of PE in the subset of patients who underwent imaging for PE.

	Had PE		Did Not Have PE		
	n	%	n	%	p
Female Sex	33	25	4696	31.61	0.1
Severe Lower Extremity Injury	49	37.12	2036	13.71	< .001
Severe Abdominal Injury	32	24.24	1043	7.02	< .001
Severe Thorax Injury	58	43.94	2805	18.88	< .001
Severe Head Injury	32	24.24	3293	22.17	0.57
Major Vascular Injury	4	3.03	217	1.46	0.14
Pelvic Fracture	31	23.48	1238	8.33	< .001
Spinal Cord Injury	5	3.79	234	1.58	0.04
Major Surgery	117	88.64	6265	42.18	< .001
Received Any Transfusion	76	57.58	2640	17.77	< .001
Received Any Red Blood Cells	70	53.03	2092	14.08	< .001
Received Plasma	27	20.45	788	5.3	< .001
Received Platelets	30	22.73	1148	7.73	< .001
>4 Transfusions in 24 hrs*	21	15.9	36	9	0.025
Tranexemic Acid*	11	8.3	18	4.5	0.091
History of VTE*	3	2.3	5	1.2	0.4
Active Malignancy*	3	2.3	9	2.2	0.99
History of Malignancy*	6	4.5	17	4.2	0.88
Increased Protime*	42	32.6	141	35.8	0.51
Enoxaparin Prophylaxis*	66	50	219	54.6	0.36
Heparin Prophylaxis*	37	28	93	23.2	0.26
No Chemical Prophylaxis*	29	22	86	21.4	0.9
Femoral Catheter*	5	3.8	24	6	0.33

Table 5 and Table 6 depict changes in demographics, injury patterns, and treatment practice between the early and late periods. 

**Table 5:** Continuous risk factors for PE in early and late periods. * Comorbidities reported in the trauma registry. ** Risk factors of PE in the subset of patients who underwent imaging for PE. PE: pulmonary embolism; BMI: body mass index; ISS: injury severity score; CTPA: CT pulmonary angiogram; PRBC: packed red blood cells; FFP: fresh frozen plasma.

	Early		Late		
	n	mean	n	mean	p
Age	7631	45.71	7245	47.17	< .001
BMI	6689	28.65	6695	28.79	0.64
ISS	7628	11.58	7245	11.87	0.09
Number of Comorbidities*	7668	0.79	7318	0.81	0.07
Greenfield Score	7668	5.52	7318	5.87	0.15
Days Until CTPA**	244	8.09	289	7.76	0.63
PRBC**	244	1.91	289	3.11	0.04
FFP**	244	0.7	289	1.58	0.04
Platelets**	244	0.61	289	0.6955	0.62
Days Without Chemical Prophylaxis**	242	3.1	288	3.2	0.77

**Table 6:** Categorical risk factors for pulmonary embolism (PE) in early and late periods. * Risk factors of PE in the subset of patients who underwent imaging for PE.

	Early		Late		
	n	%	n	%	p
Female Sex	2344	30.57	2387	32.62	0.009
Severe Lower Extremity Injury	1080	14.08	1052	14.38	0.99
Severe Abdominal Injury	510	6.65	565	7.72	0.01
Severe Thorax Injury	1582	20.63	1281	17.5	<0.001
Severe Head Injury	1757	22.91	1568	21.43	0.03
Major Vascular Injury	98	1.28	122	1.67	0.004
Pelvic Fracture	586	7.64	683	9.33	<0.001
Spinal Cord Injury	145	1.89	94	1.28	0.003
IVC Filter	44	0.57	45	0.61	0.74
Major Surgery	3054	39.83	3328	45.48	<0.001
Received Any Transfusion	1342	17.5	1374	18.78	0.043
Received Red Blood Cells	1074	14.01	1088	14.87	0.134
Received Plasma	398	5.19	417	5.7	0.17
Received Platelets	575	7.5	603	8.24	0.092
>4 Transfusions in 24 hrs*	21	8.61	36	12.46	0.15
Tranexemic Acid*	9	3.69	20	6.92	0.10
History of VTE*	4	1.64	4	1.38	0.81
Active Malignancy*	7	2.87	5	1.73	0.38
History of Malignancy*	11	4.51	12	4.15	0.84
Increased Protime*	85	35.86	98	34.27	0.70
Enoxaparin Prophylaxis*	145	59.43	140	48.44	0.01
Heparin Prophylaxis*	52	21.31	78	26.99	0.13
No Chemical Prophylaxis*	46	18.85	69	23.88	0.16
Femoral Catheter*	14	5.74	15	5.19	0.78

Table 7 shows results of multivariate analysis of independent predictors of PE.

**Table 7:** Multivariate analysis. Significant independent predictors of pulmonary embolism (PE) for all trauma patients. *Days without prophylaxis for PE workup population.

	Odds Ratio	p
Years after 2012	1.16	0.003
Severe lower extremity injury	1.93	0.001
Severe thorax injury	1.7	0.03
Major surgery	5.04	<0.001
Transfusion	2.3	<0.001
Days without chemical prophylaxis*	1.05	0.32

## Discussion

PE is a common and potentially fatal disorder that can be difficult to diagnose and manage. It is the third most common cardiovascular syndrome following myocardial infarction and stroke, responsible for 100,000 deaths annually in the United States [9]. PE is a particularly important issue in trauma. Trauma patients are at high risk for PE due to immobility, need for surgical procedures, and direct tissue damage. Furthermore, the rate of PE among trauma patients has been increasing over time. A recent study from the United Kingdom showed that 48 (4.6%) complex trauma patients were diagnosed with PE [10].

We grouped possible explanations into modifiable and non-modifiable factors. We will address non-modifiable risks first.

Increasingly sensitive technology

One possibility is that the increased PE rate is due to more sensitive testing. As CT technology advances, CTPAs are becoming more sensitive for detecting emboli. Following the introduction of CTPA in 1998, the incidence of PE in the US rose 81% with a decrease in case fatality rate [11,12]. Recently, Schultz et al. reported that 24% of patients screened with CTPA showed an incidental PE. This suggests that if we ordered enough CTPAs, incidental PE would be diagnosed in up to one-fourth of trauma patients [11]. Our data show a non-significant increase in the ratio of positive to negative CTPAs, also without worsening outcome. These data do not permit generalization, but this could be a sign of more sensitive imaging.

We evaluated this possibility by examining the anatomic distribution of PE diagnosed by CTPA. Although the clinical significance is debated, the incidence of more distal, including subsegmental, PEs is increasing in mixed inpatient populations [13]. We considered that better image quality might result in the detection of smaller and more distal pulmonary emboli in our population that previously went undiagnosed. What we found was that anatomic PE distribution was almost identical with subsegmental and isolated subsegmental PE rate diminishing slightly. This does not exclude the possibility that CTPAs are getting more sensitive, but we found no evidence of a distal migration of PE with time.

Demographic risk

Our trauma population has gotten older with more female patients and higher comorbidity. Age could account for part of the PE increase. Age has long been recognized as a risk factor for PE with an incidence 2-7 times higher in patients older than 55 [1]. Female sex is unlikely to be a contributing factor. Males are slightly more likely to develop a PE with an incidence of 56 males and 48 females per 100,000 people [2]. In spite of the nationwide obesity epidemic, our average BMI has changed minimally over the time period of this study.

Risk due to type and severity of injury

Certain injury patterns correlate with PE rate. Spinal cord injuries, traumatic brain injuries, lower extremity fractures, and severe chest injuries all predict higher likelihood of PE [11]. Head injury is less consistent [14]. Our injury pattern has changed significantly over the years, but not consistently in a direction that explains an increase in PE.

Overtesting

We considered whether the increase in PE rate might be due to a reduced threshold for ordering CTPA. A surveillance bias occurs when outcome depends on the degree of screening and detection rather than the underlying prevalence of illness. There is evidence that the nationwide increase in PE rate may be related to surveillance bias. Bilimoria et al. found that risk-adjusted VTE rates correlated inversely with adherence to VTE prophylaxis, but increased with rates of VTE imaging, suggesting that more imaging results in a greater incidence of PE [15].

Our percentage of patients undergoing CTPA is significantly higher in the later period. One explanation is that more PEs are really occurring and practitioners are responding appropriately to the clinical signs. Another possibility is that the threshold for ordering them has gone down. We cannot directly evaluate the indications for ordering a CTPA with our current data. Often, the clinical gestalt that prompts the workup is not well documented. However, we did look at indirect ways of measuring the threshold for ordering CTPA.

We compared the number of days after admission when the CTPA was ordered during the early and late periods. In this way, we use the latency of the workup as a surrogate for the threshold. If the threshold is decreasing, one would expect that on average the studies would be ordered earlier in the admission. In fact, the average time between admission and CTPA is almost identical, so it does not appear that we are quicker to initiate the PE evaluation in the later period.

If the threshold for investigating PE was getting lower, one would expect an increase in ratio of PE diagnosed by dedicated PE protocol versus incidental discovery. Conversely, if more sensitive imaging is the cause, we should be seeing more incidental PEs. We found that the rate of incidental PEs did not increase over time, pointing toward testing strategy rather than imaging sensitivity as an explanation.

Modifiable risks, preventative measures

We next asked the question: “If changing technology or changes in our patient mix is not clearly responsible, is it something we are doing or failing to do? Are we properly compliant with preventive measures?

DVT prevention

One hypothesis for the increase in PEs is that in the early period, we were finding and treating more DVTs, thus preventing them from progressing to PEs. There has been no change in our official policy, which in accordance with the 9th edition of the American College of Chest Physicians Antithrombotic Therapy and Prevention of Thrombosis Guidelines (AT9), does not include mandatory screening duplex studies [16]. Our data shows that we are finding slightly more DVTs in the later period, but the difference is not significant. This makes it unlikely that the problem is a failure to aggressively screen for DVT. We also considered whether more inferior vena cava (IVC) filters were placed during the early period, preventing DVTs from reaching the pulmonary vessels. The filter rates for the early and late periods were almost identical, thus the problem is not a reduction in the use of filters.

Mechanical prophylaxis and aggressive mobilization by physical therapy are important preventive measures for VTE. Although we are confident that compliance with mechanical prophylaxis and aggressive mobilization is high, it is difficult to quantify these variables in the chart review. The most important and often most challenging measure we can use to prevent VTE is chemical prophylaxis, which reduces VTE by 50%-80% in a variety of clinical settings [17]. There are many barriers to optimal use of VTE prophylaxis: accurate VTE risk assessment, balancing the risk of VTE versus the risk of bleeding, patient acceptance, and proper administration of prescribed therapy. These barriers often result in imperfect compliance, which represents an opportunity for improvement.

To examine the possible effect of withholding preventive measures, we recorded days without chemical DVT prophylaxis. This includes time prior to initiation and subsequent days on which it was held due to high-risk procedures, clinical bleeding, or other issues. The totals are almost identical. 3.1 versus 3.2 days. This represents an absolute difference of about two hours per patient. In our chart review, when prophylaxis is held, it is almost always due to the risk of bleeding. Although withholding prophylaxis for bleeding risk may be justified, greater than three days unprotected represents a target for improvement. All evidence-based guidelines rely on clinical judgement to balance the risks of hemorrhage and thrombosis. Refining this clinical judgement is a way to improve performance.

Differences in trauma treatment - pro-coagulant therapy

Tranexamic Acid (TXA)

Our use of TXA is at the discretion of the treating physician rather than a formal part of the MTP. Our data shows a trend toward a greater percentage of patients who received TXA in the later period, which may have had a small influence on the PE rate. The CRASH-2 trial in 2013 showed that TXA reduced mortality in bleeding trauma patients [18]. Variation in timing, dosing, and patient injury severity associated with TXA administration has contributed to conflicting information regarding the use of TXA and subsequent VTE risk in trauma patients. A retrospective review by Myers et al. suggested TXA use is associated with VTE [19]. Our data shows a trend toward a greater percentage of patients who received TXA in the later period, which may have had a small influence on the PE rate.

Hemostatic Resuscitation

Hemostatic resuscitation is a relatively new approach in which transfusion of red blood cells, plasma, and platelets in high ratios has been associated with improved survival in trauma patients [20]. Some studies have shown increased thrombosis in recipients of large quantities of blood products, for instance, the CRASH-2 study showed an association between RBC transfusion and thrombotic events including PE [18]. The prescribed ratio of PRBC to FFP to platelets of 1:1:1 in the MTP did not change over the study period. The logistics of preparing and delivering the blood products continually improved via measures such as the use of thawed plasma and coolers with the correct blood products more readily available in the trauma bay and the operating room. We found that the potential predictors of PE that differed significantly between the early and late periods were the number of patients receiving blood products and the number of units of PRBCs and FFP received. This significant finding of increased VTE risk due to exposure to blood products may have increased our PE rate and may be generalizable to other institutions.

Multivariate analysis

Years after 2012 is an independent predictor of PE rate after adjusting for potential confounders. This suggests the influence of an unmeasured risk factor or complex interaction among risk factors. Days without chemical DVT prophylaxis was associated with an adjusted odds ratio very near one and did not approach statistical significance, hence it was not an independent risk factor. We interpret this as further evidence that a change in DVT prophylaxis practice is not responsible for the increase in PE rate.

Outcomes

The death rate for patients who suffered PE is lower than for those who did not (p=0.55). Of the five deaths, only one patient died of PE. Twelve patients who suffered PE had bleeding complications on therapeutic anticoagulation, which contributed to one of the deaths. Although the PE rate in 2018 was nearly triple the PE rate in 2012, there was no increase in mortality. This led us to consider whether we are diagnosing events of low clinical significance. This is consistent with a review of the Nationwide Inpatient Sample and Multiple Cause-of-Death databases conducted by Wiener et al. which showed increasing incidence of PE without accompanying increase in mortality [12]. Therapeutic anticoagulation for PEs presenting low clinical risk could increase the risk of hemorrhage with uncertain benefit.

Between the early and late periods, there were many differences in demographics, injury patterns, and treatment practices potentially contributing to the increase in PE rate. After examination of the risk factors, we divided them into three categories:

1. Reasons not likely to account for PE increase (minimal difference or risks diminished with time): Failure to screen for and treat DVTs, not placing IVC filters, withholding chemical DVT prophylaxis, increasing obesity of the population, and shift to more distal PEs. This first group is important because it includes standard preventive measures, and hence does not identify a lapse in quality of care.

2. Possible reasons contributing to PE increase (suggested by trends in the data): changing demographics and injury patterns, more major surgery, ordering more CTPAs/lower threshold, better quality imaging, greater use of TXA.

3. Likely reason for PE increase (larger difference which may be generalizable to other centers due to statistical significance): greater likelihood of receiving massive transfusions/hemostatic resuscitation.

Limitations

There are several limitations to this study. This was a single-institution study, which can affect its generalizability. Additionally, causal relationships between risk factors and the changing PE rate could not be determined due to the retrospective and observational design. While this study includes over 14,000 patients, there was some data missing for some patients in the trauma database. This required modification of three categories of the Greenfield Risk Assessment as described in the Methods section. A study evaluating the same risk factors over time at multiple institutions could provide further insight into factors related to PE. In our institution, thromboelastography (TEG) analysis is performed routinely whenever a patient receives blood products. The influence of TEG is beyond the scope of this study, but a future study could correlate TEG parameters with subsequent thrombotic complications.

## Conclusions

The advent of the hemostatic resuscitation paradigm in our institution was associated with an increase in PE rate. Balanced resuscitation promotes coagulation, but it could also cause coagulation where it is not desired, leading to thrombotic complications. It is important to recognize the unintended consequences of new therapy. Hemostatic resuscitation is the factor most likely to have contributed to our rising PE rate, however, PE in trauma is multifactorial and complications may increase even when there is no measurable decrease in quality of care. We advise caution against overreliance on PE and other simple indices as measures of quality. Given the overall increase in VTE in the trauma population, many individual hospitals are likely to see their PE rates increase. A goal of this paper is to offer a model of how to respond to an increase in a complication. Even if a potential risk factor could have happened by chance, if it is consistent with current standard of care, it is a suitable target for quality improvement. Institutional responses to our increase in PE rate include attempting to miss fewer days of prophylaxis while continuing to balance the risk of bleeding and thrombosis, standardizing indications for CTPA without eliminating clinical judgement, encouraging evaluation of pre-test risk by a practitioner with a higher level of training such as a chief resident or attending physician, and increasing use of a pre-test risk estimation tool such as the Wells score. By these means, we hope to reverse our trend of increased PEs regardless of cause.

## References

[1] Bahloul M, Dlela M, Bouchaala K, Kallel H, Ben Hamida C, Chelly H, Bouaziz M (2020). Post-traumatic pulmonary embolism: incidence, physiopathology, risk factors of early occurrence, and impact outcome. A narrative review. Am J Cardiovasc Dis.

[2] Giordano NJ, Jansson PS, Young MN, Hagan KA, Kabrhel C (2017). Epidemiology, pathophysiology, stratification, and natural history of pulmonary embolism. Tech Vasc Interv Radiol.

[3] Anderson FA Jr, Spencer FA (2003). Risk factors for venous thromboembolism. Circulation.

[4] Jacobs BN, Cain-Nielsen AH, Jakubus JL, Mikhail JN, Fath JJ, Regenbogen SE, Hemmila MR (2017). Unfractionated heparin versus low-molecular-weight heparin for venous thromboembolism prophylaxis in trauma. J Trauma Acute Care Surg.

[5] Geerts WH, Bergqvist D, Pineo GF, Heit JA, Samama CM, Lassen MR, Colwell CW (2008). Prevention of venous thromboembolism: American College of Chest Physicians evidence-based clinical practice guidelines (8th edition). Chest.

[6] Gidwani R, Bhattacharya J (2015). CMS reimbursement reform and the incidence of hospital-acquired pulmonary embolism or deep vein thrombosis. J Gen Intern Med.

[7] (2021). Preventing hospital-associated venous thromboembolism. https://www.ahrq.gov/patient-safety/resources/vtguide/index.html.

[8] Greenfield LJ, Proctor MC, Rodriguez JL, Luchette FA, Cipolle MD, Cho J (1997). Posttrauma thromboembolism prophylaxis. J Trauma.

[9] Konstantinides SV, Meyer G, Becattini C (2019). 2019 ESC Guidelines for the diagnosis and management of acute pulmonary embolism developed in collaboration with the European Respiratory Society (ERS). Eur Respir J.

[10] Glover TE, Sumpter JE, Ercole A (2019). Pulmonary embolism following complex trauma: UK MTC observational study. Emerg Med J.

[11] Schultz DJ, Brasel KJ, Washington L (2004). Incidence of asymptomatic pulmonary embolism in moderately to severely injured trauma patients. J Trauma.

[12] Wiener RS, Schwartz LM, Woloshin S (2011). Time trends in pulmonary embolism in the United States: evidence of overdiagnosis. Arch Intern Med.

[13] Carrier M, Klok FA (2017). Symptomatic subsegmental pulmonary embolism: to treat or not to treat?. Hematology Am Soc Hematol Educ Program.

[14] Jeremitsky E, St Germain N, Kao AH, Ong AW, Smith RS (2013). Risk of pulmonary embolism in trauma patients: not all created equal. Surgery.

[15] Bilimoria KY, Chung J, Ju MH, Haut ER, Bentrem DJ, Ko CY, Baker DW (2013). Evaluation of surveillance bias and the validity of the venous thromboembolism quality measure. JAMA.

[16] Guyatt GH, Akl EA, Crowther M, Gutterman DD, Schuünemann HJ (2012). Antithrombotic therapy and prevention of thrombosis, 9th ed: American College of Chest Physicians evidence-based clinical practice guidelines. Chest.

[17] Nicholson M, Chan N, Bhagirath V, Ginsberg J (2020). Prevention of venous thromboembolism in 2020 and beyond. J Clin Med.

[18] Perel P, Clayton T, Altman DG (2014). Red blood cell transfusion and mortality in trauma patients: risk-stratified analysis of an observational study. PLoS Med.

[19] Myers SP, Kutcher ME, Rosengart MR, Sperry JL, Peitzman AB, Brown JB, Neal MD (2019). Tranexamic acid administration is associated with an increased risk of posttraumatic venous thromboembolism. J Trauma Acute Care Surg.

[20] Bogert JN, Harvin JA, Cotton BA (2016). Damage control resuscitation. J Intensive Care Med.

